# The genomic sequence of packaged viral ribonucleic acid predicts mucosal HIV-1 transmission fitness

**DOI:** 10.1016/j.isci.2026.116712

**Published:** 2026-07-13

**Authors:** Rahul Pawa, Iulian Derecichei, Katja Klein, Muhammad Ali, Chanuka N. Wijewardhana, Richard Gibson, Ryan M. Troyer, Robin J. Shattock, Eric J. Arts, Jamie F.S. Mann

**Affiliations:** 1 Department of Microbiology and Immunology, University of Western Ontario, London, ON N6A 5C1, Canada; 2 Department of Infectious Diseases, Division of Medicine, Imperial College London, Norfolk Place, London W2 1PG, UK; 3 Joint Clinical Research Center, Kampala, Uganda; 4 Bristol Veterinary School, University of Bristol, Bristol BS40 5DU, UK

**Keywords:** HIV, transmission, viral evolution, replication, innate immunity

## Abstract

RNA viruses are responsible for many zoonotic disease transmission events, and remain a global health challenge. To evade immune detection, RNA viruses suppress the numbers of immunostimulatory oligonucleotide motifs (INMs) present in their genomes. Although this is thought to occur in human immunodeficiency virus-1 (HIV-1), our bioinformatic analysis on well characterized clinical datasets, along with *in vitro* studies, demonstrate that HIV-1 is enriched for INMs within its transmitted/founder (T/F) population relative to non-transmitting variants. Importantly, our data suggests that within the host, there is an evolutionary genetic replacement of T/F viruses that otherwise exhibit high transmission fitness and low replicative fitness, with variants having low transmission fitness but high replicative fitness. These findings provide insights into HIV-1 transmission by studying within-host and between-host evolutionary dynamics, enabling us to identify a framework for HIV infection biology, with viral RNA playing a role in transmission and replication processes.

## Highlights

• Transmitted HIV-1 virus contains elevated numbers of RNA immunostimulatory motifs
• Transmitted HIV-1 virus induces higher IRF activation than chronic viruses
• HIV-1 genomes exhibit biased codon usage, which is linked to viral fitness

## Graphical abstract

**Figure:**
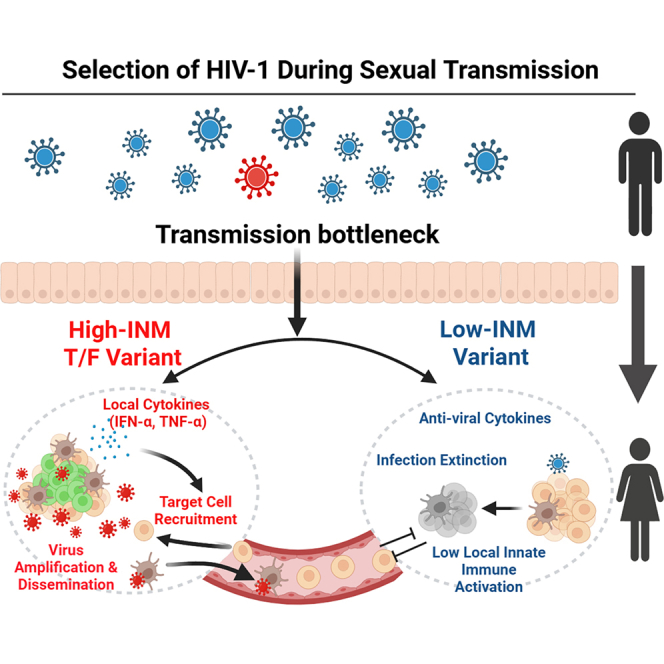

## Teaser

Immunity; virology; genomics

## Introduction

Human immunodeficiency virus type 1 (HIV-1) is the rapidly mutating RNA-based retrovirus behind the global acquired immunodeficiency syndrome (AIDS) epidemic. HIV-1 is spread primarily through sexual intercourse,^1^ with the virus passing through the mucosal barrier and infecting CD4 T cells of the Th17 phenotype.^2^ While the recipient is exposed to thousands of HIV-1 variants from the donor, in 70%–80% of infections, only a single virus (called the transmitted/founder; T/F) successfully overcomes a stringent genetic bottleneck to establish infection within the recipient.^2^^,^^3^^,^^4^ Several studies have suggested that viral genetic and phenotypic traits might select for T/Fs during the transmission process.^5^^,^^6^^,^^7^^,^^8^ Such selection biases, if identifiable, would be critical for the development of an effective HIV vaccine.

To date, many studies on T/F HIV-1 have concentrated on the structural and functional traits of the HIV-1 envelope (Env) protein, a surface glycoprotein characterized by high genotypic and phenotypic diversity. T/F Envs have been described as having unique characteristics when compared to viruses isolated in late infection.^9^ For example, when looking at glycosylation, T/Fs have fewer potential N-linked glycosylation sites (PNLGs).^7^^,^^9^^,^^10^ This lack of PNLGs is likely due to their shorter V1/V2 loops and is thought to aid the virus in crossing the lectin-rich vaginal mucosal barrier.^7^ Furthermore, HIV-1 with T/F Env had higher transmission fitness in assays involving cervical and penile tissue explants,^6^ which may in turn be associated with Env glycan composition on T/F Env.^8^ However, the impact of the viral RNA genome in enabling T/F variants to successfully outcompete other variants within the genetic bottleneck has received comparatively little attention. The HIV-1 genome consists of two copies of single stranded RNA, a known and potent inducer of intracellular pattern recognition receptors (PRRs), such as Toll-like receptors (TLR) 7, TLR8,^11^ and the Retinoic acid-inducible gene I (RIG-I)-like receptors via structured RNA.^12^ The role of immunostimulatory oligonucleotide motifs (INMs) in the HIV-1 RNA genome during transmission is not clear, including the ability and extent that different HIV-1 variants activate PRRs, and whether this influences the selection of T/F viruses within the mucosa. Although direct data on HIV INMs are lacking, studies in other RNA viruses indicate that modest changes in INM frequency can quantitatively alter innate immune activation. This is best demonstrated for CpG motifs, which are selectively depleted during RNA virus adaptation to humans. In influenza A virus, increasing CpG numbers in a single viral segment enhances type 1 interferon secretion by plasmacytoid dendritic cells (DCs) via, showing that small changes in motif number can measurably affect innate sensing.^13^^,^^14^ By analogy, subtle variation in other immunostimulatory motifs, including those in HIV-1, may similarly modulate PRR activation during early infection.

Upon Env interaction with CD4 and CCR5 triggering cell-viral membrane fusion and expulsion of the core into the cytoplasm, the HIV-1 RNA genome remains protected within the viral capsid, a covering that’s relatively impervious to PRR surveillance. Furthermore, even foreign DNA sensing PRRs, e.g., interferon-inducible protein 16 (IFI16) and cyclic guanosine monophosphate (GMP)-AMP synthase (cGAS) may not have access to reverse transcribed HIV-1 DNA, considering that the capsid remains relatively intact during its translocation to the nucleus for proviral DNA integration.^15^ Nonetheless, the host has adopted multiple innate antiviral measures in attempts to disrupt viral assembly and core stability, e.g., TRIM, SERINC5, APOBEC3G, BST2, and SAMHD1, which promotes viral RNA recognition by PRRs.^16^ PRRs such as TLR7 and TLR8 are more likely to be activated following endocytosis and subsequent capsid breakdown in endosomes, particularly when viral entry occurs without vesicle-virus membrane fusion. In the case of primary infection of, and transmission through mucosal tissues, various tissue-resident myeloid cells (macrophages and DCs) acting as antigen-presenting cells (APCs) may be the first to encounter, endocytose/phagocytose, and process HIV-1, leading to exposure of viral genomes to these PRRs. Collectively suggesting that temporal and spatial properties maybe at play during innate recognition of HIV-1 viral RNA.

Shortly after transmission, foci of infection (clusters of infected cells) are established in the mucosa before the virus disseminates to the local draining lymph nodes.^17^ Since susceptibility to HIV-1 is significantly enhanced in individuals with ongoing inflammation,^18^ we asked whether the virus itself could enhance transmission success by triggering innate, intracellular immune responses. Specifically, we asked whether individual viral RNA genomes in the quasi-species may differ in the number of INMs with PRR-stimulating potentials, and if their ability to activate immune responses could be a purifying factor to select for the T/F HIV-1 from the inoculating HIV-1 population. Considering HIV-1 RNA genomes can stimulate innate PRRs, and because TLR-induced maturation of APCs, such as DCs, facilitates their migration from sites of infection,^19^ we hypothesized that HIV-1 T/F viruses might use activation of PRRs to outcompete the other variants in the inoculating quasi-species.

To begin to address the role of the HIV-1 genomic RNA on establishing new infections, we performed a genetics-based epidemiological examination of multiple well-characterized human clinical datasets (Figure S1), along with analysis of published *in vitro* datasets. By analyzing thousands of HIV-1 sequences, we identified an intriguing biological association with the numbers of known INMs and successful viral transmission. Specifically, we found HIV-1 viruses with a greater number of INMs were significantly more successful in the transmission process. Furthermore, these viruses with higher INMs were more prevalent within the semen of donors compared to donor blood. Interestingly, viruses with enhanced transmission fitness, had significantly elevated numbers of INMs compared to viruses isolated at chronic stage of HIV-1 infection, with the former having reduced replication fitness compared to chronic viruses. This suggests an evolutionary switch between transmission and replicative fitness is at play and that the immunostimulatory nature of HIV RNA genomes could have a central role in these processes.

## Results

### Quantifying specific oligonucleotide motifs within viral genomes demonstrates T/F viruses have elevated numbers of immunostimulatory sequences

During sexual transmission, HIV-1 enters the host and is immediately faced with a mucosal barrier, used in part by the host to discriminate between foreign and endogenous antigens. Therefore, early innate detection systems, such as PRRs, play crucial roles in the transmission and infection process. We used a computational approach to predict the potential antigenicity of HIV-1 variant RNAs by identifying and counting single-stranded RNA sequence motifs in the various regions of HIV-1 genomic RNA. The INMs selected for counting are known to stimulate TLR7/8 and induce TNF-α and/or IFN-α secretion from cells.^20^ The overall prevalence of these INMs in HIV-1 subtype B reference strains obtained from the Los Alamos National Laboratory (LANL) HIV sequence database (Figure S2C) was 4.1% of the total genome length but INMs were not uniformly distributed in the cataloged genomes, suggesting the presence of HIV-1 genes/regions with enhanced PRR stimulatory potential. The *env* gene, compared to all other coding regions, contained the greatest number of INMs (mean = 122 motifs) and the highest density (4.8% of the total gene length) in the HIV-1 genome. In contrast, the predicted pro-inflammatory CpG motifs were comparably fewer, comprising only 1.7% of the genome, with its greatest concentration in the *nef* gene (1.3% of the *nef* sequence) and only 1% of the *env* gene sequence (Figure S2C). Based on this preliminary data, we proceeded with our cohort analyses, focusing on studying INMs in the *env* gene.

**Figure 1:**
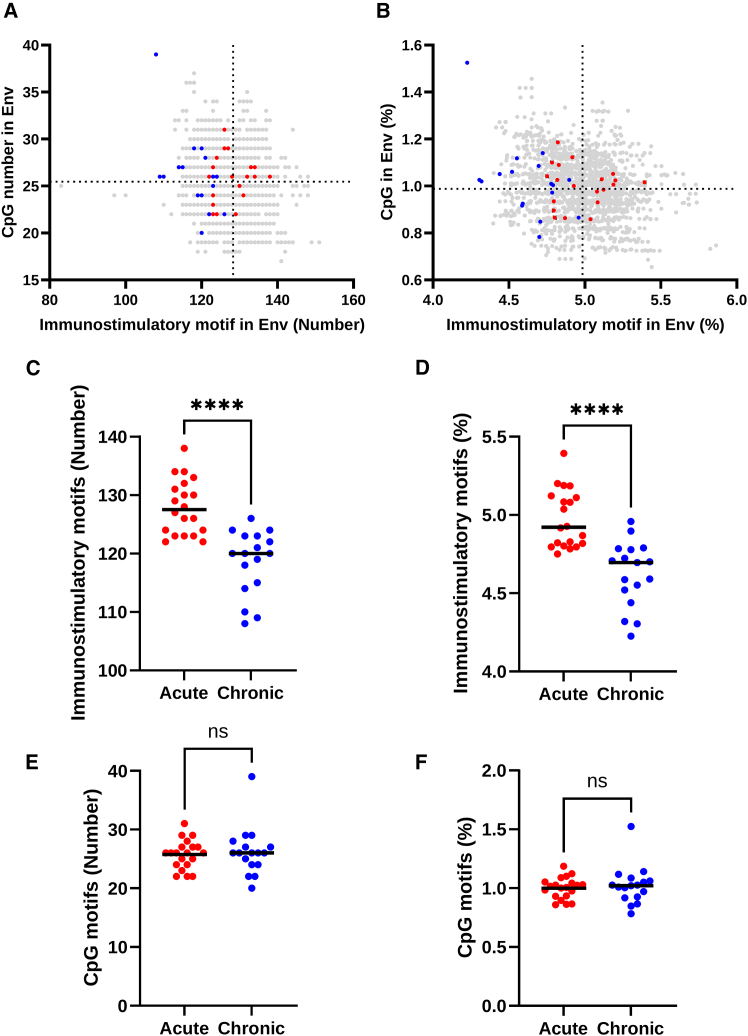
Known HIV T/F virus sequences do have elevated numbers of INMs and cluster together HIV-1 subtype B *env* sequences were downloaded from LANL (*n* = 8,040), aligned to HXB2 and trimmed according to HXB2 *env*. The (A) numbers and (B) percentages of INMs and CpG motifs were quantified and graphically represented as scatterplots. To determine if T/F viruses distribute evenly within the LANL sequences or if they cluster together within a defined area of the scatterplots, the INMs and CpG motif numbers (red dots) from known T/F virus *env* sequences from CHAVI and the HIV Reagent Program were superimposed onto the LANL sequences (gray dots) and compared to chronic viruses from patients infected for greater than a year (blue dots). Plots representing INMs as both (C) number and (D) percentage of *env* between T/Fs and chronic viruses. Plots representing CpG as both (E) number and (F) percentage of *env* between T/Fs and chronic viruses. Statistical significance was assessed using a non-parametric Mann-Whitney test for comparisons of mean rank values among the different groups (ns, not significant; ∗∗∗∗*p* ≤ 0.0001).

**Figure 2:**
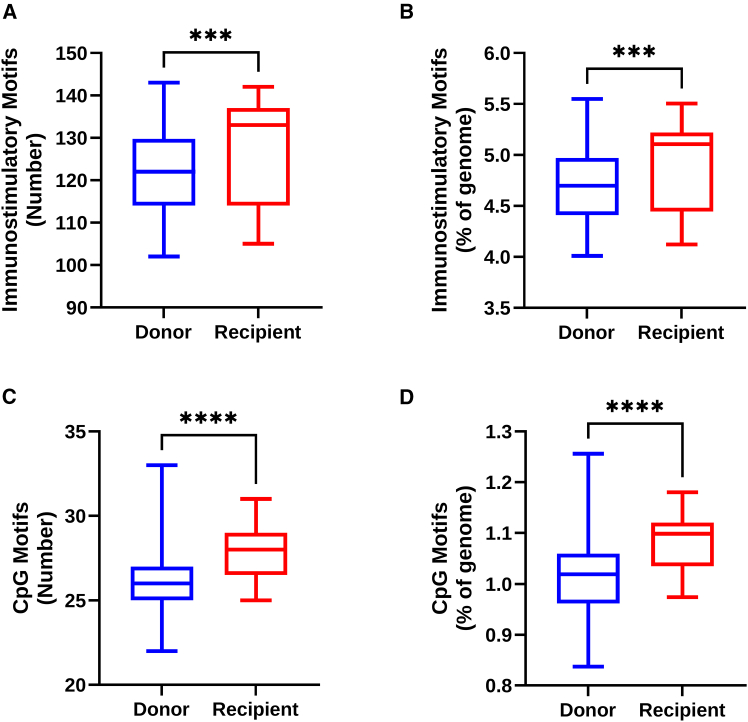
The recipients within transmission pairs have viral genomic sequences with higher numbers of immunostimulatory RNA motifs compared to their respective donors Donor and recipient HIV-1 sequences, attained from Iyer et al.,^5^ were aligned based on HXB2 and trimmed such that all *env* sequences were the same length. All donor and recipient sequences were analyzed for the (A) numbers and (B) percent of INMs (C) numbers, and (D) percent of CpG motifs. Statistical significance was assessed using a non-parametric Mann-Whitney test for comparisons of mean rank values among the different groups (∗∗∗*p* ≤ 0.005, ∗∗∗∗*p* ≤ 0.0001). Data are shown as box-and-whisker plots (line = median, whiskers = minimum and maximum values).

Knowing that INMs sequences are more abundantly found in the *env* gene compared to any other HIV coding region we asked the question, do T/F HIV-1 Envs have more INMs within their *env* genomes than those viruses obtained at chronic stage of infection? To address this question, we plotted the number of INMs in (1) 8,040 LANL *env* sequences from subtype B viruses obtained at all (or unknown) times post-infection (months to years) (gray dots, Figures 1A and 1B), (2) chronic viruses from patients infected for greater than a year (blue dots), and superimposed (3) the number of INMs of T/F subtype B *envs* (red dots) (from Center for HIV-1/AIDS Vaccine Immunology and the HIV Reagent Program).^21^ As shown, the *env* sequences (from unknown time lengths of infection) were dispersed between a range of 83 and 151 INMs (median = 128.3) and 17 and 39 CpG motifs (median 25.4). The T/F *env* sequences had greater numbers (128 vs. 118.6, ∗∗∗∗*p* < 0.0001) and density (4.97% vs. 4.62%, ∗∗∗∗*p* < 0.0001) of INMs than chronic *env’s* but similar number of CpG motifs (Figures 1C–1F), all of which fell within the expected range of INMs in the overall subtype B population (Figures 1A and 1B). This difference in the number of INMs between T/F and chronic viruses might suggest different immune stimulatory potential by genomic RNA in T/F HIV-1 compared to that in chronic HIV-1. If true, INMs in the *env* gene might be a predictive biomarker for viruses, which are likely to exhibit greater transmission fitness.

### HIV-1 variants with increased numbers of INMs are more likely to be transmitted to the recipient

To determine if the number of INMs in viral genomes has any impact on transmission success, we analyzed 282 HIV-1 *env* sequences^5^ from blood samples of donor and recipient pairs at near the time of transmission. The HIV *env* RNA sequences identified in the recipients had significantly greater numbers of INMs (∗∗∗*p* < 0.0005) (Figures 2A and 2B) and CpG (∗*∗∗∗p* < 0.0001) (Figures 2C and 2D) motifs compared to their respective donors. We further confirmed this study by analyzing the virus sequences in a second transmission pair cohort (Figure S3).^22^ Collectively, these data suggest a selection bias in the variants that establish infection in the recipient’s blood compared to the HIV-1 population found in the donor. Specifically, the donor harbors a diverse HIV-1 population with the HIV-1 variants containing high numbers of INMs outcompeting those with low numbers of INMs to establish infection in the recipient.

### Virus with greater numbers of INMs in their genomes is more likely to be found in the donor’s semen compared to their blood

**Figure 3:**
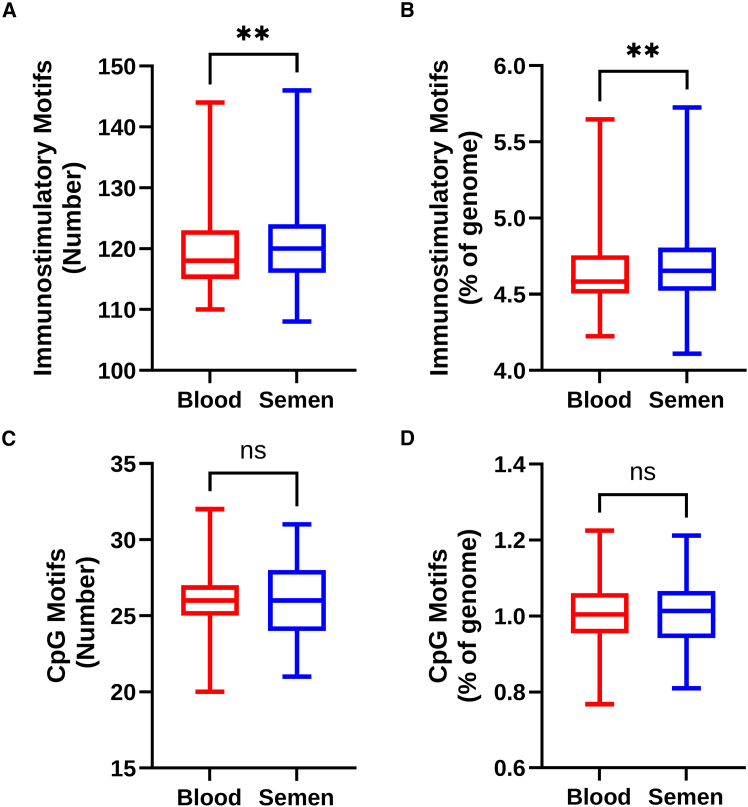
HIV-1 virus in semen contains viral variants with genomes containing higher numbers of INMs compared to variants in the blood compartment Matched blood vs. semen viral sequences were attained from Anderson et al.^23^ The *env* sequences were analyzed from 14 volunteers. All semen and blood sequences were analyzed for the (A) numbers and (B) percent of INMs and (C) numbers, and (D) percent of CpG motifs. Statistical significance was assessed using a non-parametric Mann-Whitney test for comparisons of mean rank values among the different groups (ns = not significant; ∗∗*p* ≤ 0.005). Data are shown as box-and-whisker plots (line = median, whiskers = minimum and maximum values).

In cases of HIV-1 transmission following heterosexual coitus or among men who have sex with men, HIV-1 variants in the donor’s semen transmitted to recipient’s vaginal or rectal tract may differ in their immunostimulatory properties from variants transmitted from donor to recipient blood. This underscores the importance of investigating viral populations in both blood and semen of HIV^+^ individuals to better understand how INMs contribute to transmission dynamics. We therefore investigated the *env* sequences of HIV-1 in semen and blood of subtype B and C infected men (Figure 3).^23^ As described earlier, *env* sequences contain the highest number and percentage of INMs within the genome of HIV-1 subtype B (Figure S2C). A similar pattern is observed in the *env* genes of subtype C HIV-1 genomes (obtained from LANL) (Figure S2D), which contains the highest number, but not the highest percentage of INMs in *env*. The *env* genes from circulating HIV-1 variants in the donors’ semen had even higher numbers of INMs (*∗∗p* ≤ 0.005) compared to those in the donors’ blood (Figures 3A and 3B). Unlike the INMs between donor and recipients, the slight increase in CpG motifs between semen and blood were not significantly different (*p* = 0.0786) (Figures 3C and 3D). This result suggests that HIV compartmentalized in the semen may have already evolved an *env* gene with higher numbers of INMs in the male reproductive organ. Semen is a primary medium of heterosexual HIV-1 transmission from male penis to rectal tract or female genital tract. This work adds to previously published literature, which suggests that the compartmentalization of the virus between semen and blood within individuals may exist; although other studies have suggested the differences observed between semen and blood are due to sampling artifacts or transient viral dynamics, rather than true compartmentalization.^23^^,^^24^^,^^25^^,^^26^^,^^27^^,^^28^^,^^29^

### HIV-1 variants with greater numbers of INMs in their genomes are more likely to establish infection within female recipients

**Figure 4:**
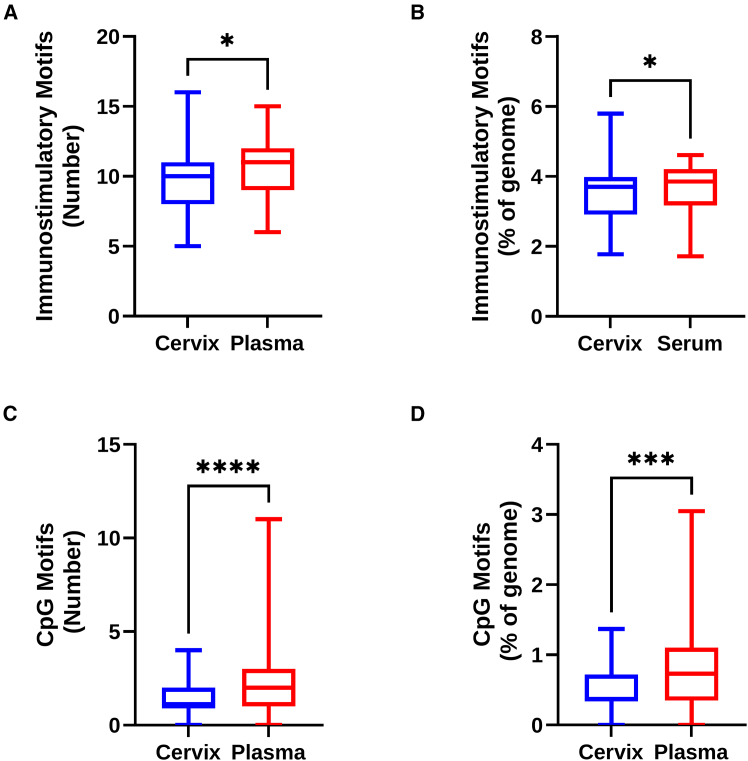
The serological compartment contains viral isolates with a higher number of INMs compared to the cervix Viral sequences from matched cervical swab and blood samples were attained from Klein et al.^30^ This cohort analyzed *env* sequences from the C2-V3 region only. All sequences within blood and mucosal samples were analyzed for the (A) numbers and (B) percent of INMs and (C) numbers, and (D) percent of CpG motifs. Statistical significance was assessed using a non-parametric Mann-Whitney test for comparisons of mean rank values among the different groups (∗*p* ≤ 0.05, ∗∗∗*p* ≤ 0.005, ∗∗∗∗*p* ≤ 0.0005). Data are shown as box-and-whisker plots (line = median, whiskers = minimum and maximum values).

During the transmission event from male reproductive tract to female genital tract, only a fraction of HIV-1 will establish infection in the female genital tract. Of that, only one HIV-1 clone, in the majority of cases, appears to translocate and establish a systemic infection characterized by the HIV-1 in blood at acute infection.^2^ As described previously, samples from blood and semen from potential donors are available and have been analyzed (Figure 3). Unfortunately, complete sample sets from donor blood, donor semen, recipient vaginal/cervical tissue, and recipient blood from heterosexual transmission pairs are not available. To further study the potential role of INMs in the HIV-1 transmission process, we analyzed the viral *env* sequences immediately following infection in female recipients, with virus sampled with a cervical swab taken during the acute/early stage of HIV-1 infection (Figures 4A and 4B).^30^ Previously, we reported that female recipients were infected with small HIV-1 populations in the cervix and suggested a subsequent selection for a single HIV-1 clone to establish systemic infection. Although no donor samples (semen or blood) were available for analysis, sequences in the recipients’ cervical swabs and the blood during early infection could be analyzed for the presence of INMs. As shown, a significantly greater number of INMs (∗*p* ≤ 0.05) (Figure 4A) and CpG motifs (∗∗∗*p* ≤ 0.005, ∗∗∗∗*p* ≤ 0.0005) (Figure 4B) were detected in the blood of recipients compared to the sequences analyzed from their matched cervical swabs, both at the time of infection. This result suggests that an HIV-1 variant with a higher number of INMs may have outcompeted the other HIV-1 variants within the inoculating swarm, to establish infection in the female genital tract. It should be noted that CpG differences were more pronounced than INMs in this dataset; however, as the analysis is limited to the C2-V3 region and not the whole *env* gene, resolution is likely reduced and these findings are interpreted cautiously.

Unlike all previous analyses, which involved measures of the full HIV-1 *env* region (∼2,600 bp), this dataset (Figure 4) only performed analysis on the viral *env* C2-V3 region, representing only a snapshot of the *env* gene (∼480 bp). We therefore performed a reanalysis of the previous datasets trimmed only to the C2-V3 region and found, with the exception of the Anderson dataset, the trend remained true with statistical significance being measured (Figure S4). This clearly demonstrates that despite a minimal region being analyzed, that INMs still differentiate T/F viruses from chronic viruses.

### T/F viruses with more INMs are more likely to be found within the recipients’ migratory cells and therefore establish systemic infection

**Figure 5:**
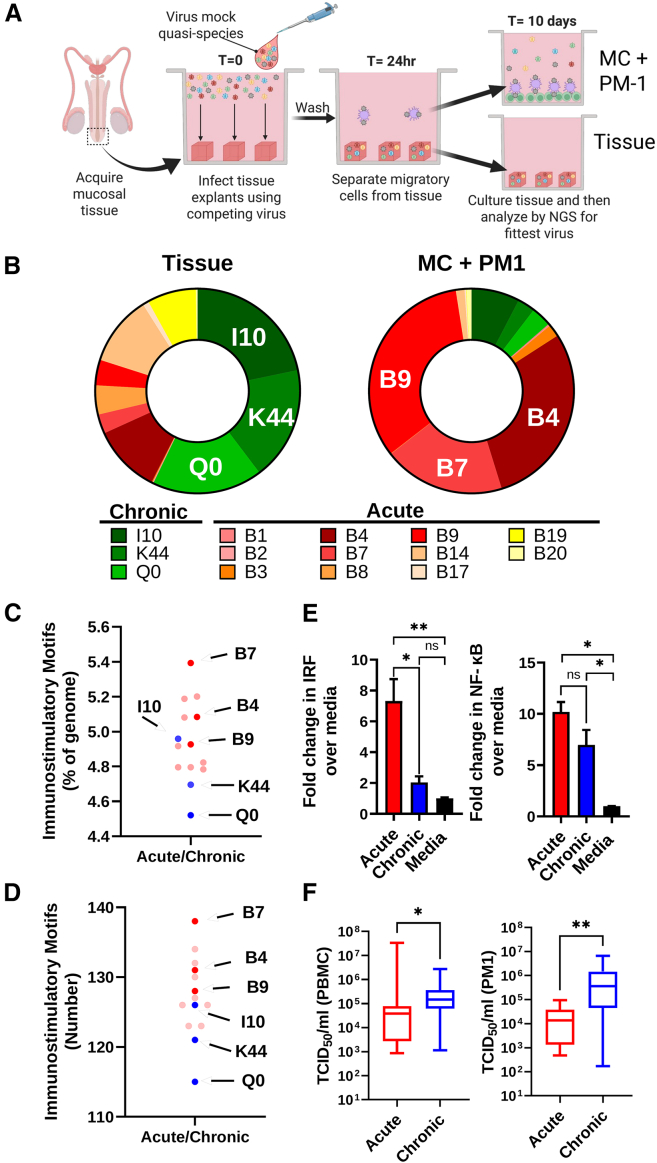
HIV-1 pseudoviruses with high number of INMs outcompete the quasi-species in human mucosal explant tissues Penile glans tissue was used to make explants, which were then infected by a mock quasispecies consisting of pseudoviruses. (A) After 10 days of culture, the tissue was analyzed by NGS and migratory cells were cocultured with PM1 cells to amplify virus and sequenced by NGS. (B) Donut charts represent the composition (%) of acute/early and chronic viruses, based on the *env* gene inserted into a common NL4-3 backbone vector, in tissue and in MC + PM1 cocultures. The total (D) number and (C) percent of INMs of acute/early (red shades) and chronic viruses (blue) is shown. (E) The fold change in viral *env* gene induced IRF activation and NF-kB activation is shown. (F) Replication of subtype B Env pseudoviruses in peripheral blood mononuclear cells (PBMCs) and in the CD4 T (PM1) cell line were compared by extracting the data from King et al.,^21^ as relative TCID_50_/mL. Statistical significance was assessed using a non-parametric Mann-Whitney test for comparisons of mean rank values among the different groups (ns = not significant; ∗*p* ≤ 0.05, ∗∗*p* ≤ 0.005, ∗∗∗*p* ≤ 0.0005). Data are represented as mean + SEM or as box-and-whisker plots (line = median, whiskers = minimum and maximum values).

Based on the prior analyses from multiple cohorts of transmission pairs and different anatomical compartments, we hypothesized that successful transmission favors virus with a high number of INMs in their genomes. With each transition from donor to recipient, compartmental bottlenecks may further enrich for the T/F HIV-1 carrying the maximal number of INMs. To test this hypothesis, we re-analyzed viral infection studies using a mixture of HIV-1 isolates (e.g., a mock quasi-species) and human mucosal explant tissue (i.e., model transmission assay).^30^ We opted for using *in vitro* human mucosal explant tissue studies, as it more accurately represents events happening during the early stages of natural transmission within intact human tissues, compared to humanized mouse models or non-human primates. Importantly, this assay approximates HIV transmission by (1) utilizing tissue from a site of natural exposure, (2) preserves the natural distribution of tissue-resident cells and enables their interactions, and (3) supports HIV infection.^31^^,^^32^ Within the mock quasi-species, we competed chimeric HIV-1 expressing different Env glycoproteins from primary isolates obtained from acute infections (B1-B20) and Env glycoproteins from chronic-stage HIV-1 infection (I10, K44, and Q0).^6^ All chimeric HIV-1 had the same common NL4-3 backbone, ensuring any effects seen in the assay were due to the inserted *env* gene. After pulsing the tissue with the competing chimeric HIV-1 quasi-species, the tissues were washed and incubated overnight, after which migratory cells (MC) were cultured for 10 days in the presence of infection permissive PM1 cells. The washed explant tissues were also cultured for 10 days. The replicating virus in the migratory cell co-cultures and within the tissues were analyzed by next-generation sequencing (Figure 5A). For these studies, we re-analyzed this *ex vivo* transmission fitness study^6^ to first establish the order of transmission fitness and then to compare against the number of INMs in their *env* gene. All the viruses replicated in the tissue explants, with the chronic chimeric HIV-1 I10 (29%), K44 (24.3%), and Q0 (23.6%) *env* being the predominant viruses detected, but these viruses were minimally associated with MC exiting the tissue (15.5%, 5.6% and 6.5%, respectively). Interestingly, acute chimeric HIV-1 expressing B4 (59%), B7 (39%), and B9 (67%) *env* were consistently the viruses found exiting the tissues associated with migratory cells (Figure 5B). Notably, these same chimeric viruses carried *env* genes with the greatest number of INMs (Figures 5C and 5D). This enrichment suggests that association with migratory cells may represent a key mechanism by which INM-rich HIV-1 variants disseminate from mucosal sites of infection to draining lymph nodes.

To segregate *env* gene and Env glycoprotein function from the underlying INMs impact on PRRs, the *env* RNA of T/F B4, B7, B9, and of chronic I10, K44, and Q0 was *in vitro* T7 transcribed, then delivered to a THP-1 reporter cell line using cationic lipid dioleoyl-3-trimethylammonium propane (DOTAP) (Figure 5E). We observed a significantly stronger activation of the interferon regulatory factor (IRF) response (transcription factors associated with the type 1 interferon response) with the T/F *env* RNA than with chronic *env* RNA. Notably, this enhanced IRF response occurred despite comparable activation of NF-kB transcription factor between the acute and chronic viral RNAs (Figure 5E). It is important to note DOTAP forms stable complexes with RNA, known as lipoplexes, which are primarily internalized via endocytosis. This property makes DOTAP particularly well-suited for studying endosomal delivery and the activation of endosomal PRRs such as TLR7. Overall, this demonstrates that viral transmission fitness is likely impacted by innate PRR activated by the viral genome and that HIV-1 viruses that successfully outcompete the remainder of the viruses in the quasi-species have elevated numbers of INMs.

Throughout these studies, we have focused on HIV-1 transmission fitness, revealing that T/F viruses migrate out of genital tissue more efficiently than chronic viruses, contain more INMs in their genomes and induce more IRF in reporter cell lines. Therefore, we asked the question, do T/F viruses also exhibit greater replication fitness compared to chronic viruses? To address this question, we reanalyzed the data from King et al.,^21^ comparing 11 T/F acute to 14 chronic viruses in different cell types. An advantage of using this dataset is that the same chimeric viruses were used as described in our analyses previously. As shown in a viral growth assay, when the T/F acute viruses were grown and compared to chronic viruses in PM1 (CD4 T cell line) and peripheral blood mononuclear cells (PBMCs), they exhibited significantly reduced replication fitness (Figure 5F). This result suggests that although HIV-1 T/F viruses clearly display enhanced transmission fitness, they have significantly reduced replication fitness, indicating that the two temporally distinct variant populations exhibit differing transmission and replication fitness’s. It should be noted that replication fitness was assessed *in vitro* in PM1 cells and activated PBMCs, which may not fully predict *in vivo* fitness in humans.

### T/F viruses possess a higher number of “high-risk” leucine codons

Up to this point, we observed that INMs were significantly more abundant in T/F viruses compared to chronic viruses. These motifs were found in the genomes of variants infecting recipients and were also enriched in virus populations in semen compared to those in blood. To further investigate, we attempted to map the location of these nucleotide motifs within the *env* genome to determine if their distribution is correlated with structural or functional domains of Env. However, no consistent patterns in their genomic localization were observed within our alignments.

**Figure 6:**
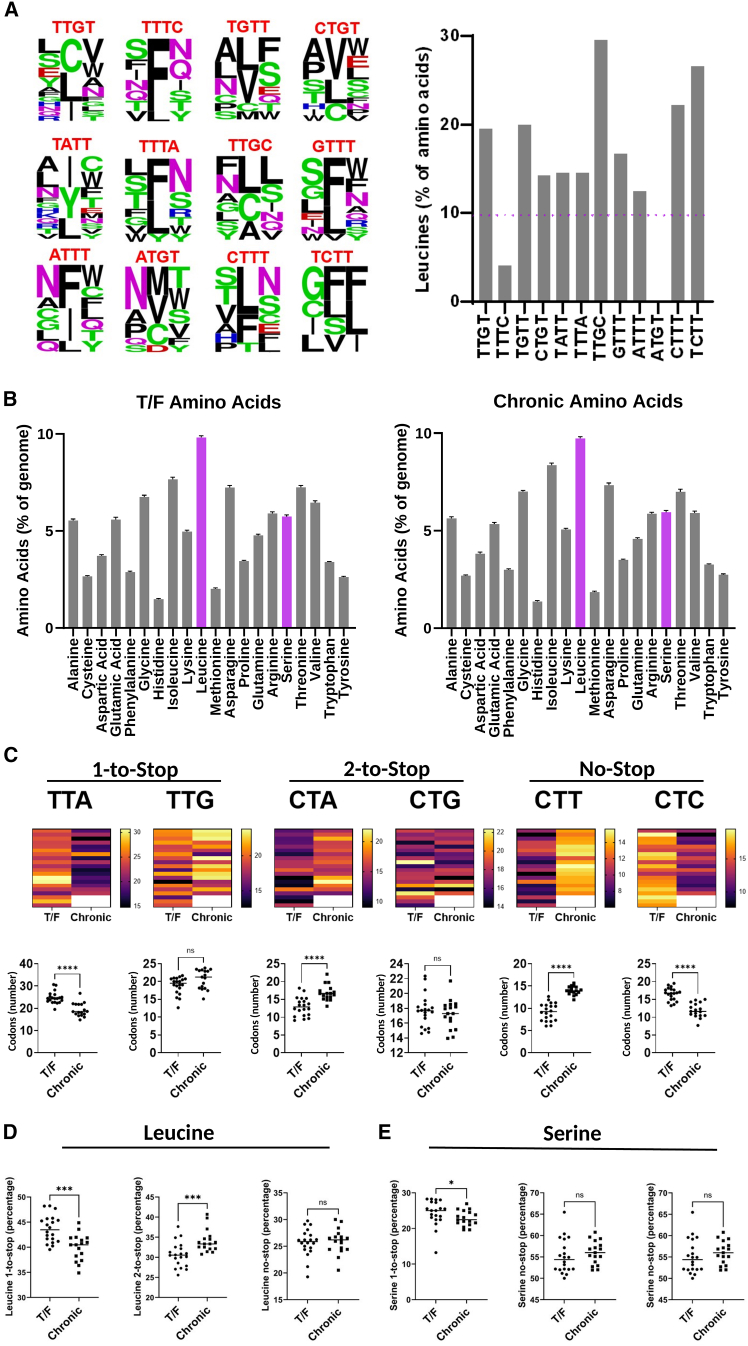
T/F viruses have a higher usage of high-risk codons Total genomes from T/Fs and chronic viruses were analyzed for amino acid frequencies and codon usage. (A) WebLogo generated from amino acid sequences extracted in a motif-centered manner across a single viral *env* genome (left) and the percentage of leucine residues across all motif-associated sequences extracted (right). (B) Amino acid frequency (% of *env* genome) for both T/F and chronic viruses. (C) The leucine codon usage frequency between T/F and chronic viruses visualized as heatmaps and corresponding dot-plots. (D) Leucine codons were stratified into high-risk 1-to-stop codons, which can create an early stop codon with a single mutation and lower-risk 2-to-stop and no-stop codons, which requires at least two unique mutations to produce a stop codon. (E) Serine codons were also stratified into high-risk 1-to-stop, 2-to-stop and no-stop. Statistical significance was assessed using a non-parametric Mann-Whitney test for comparisons of mean rank values among the different groups (ns = not significant; ∗*p* ≤ 0.05, ∗∗∗*p* ≤ 0.005, ∗∗∗∗*p* ≤ 0.0005). Data are represented as mean + SEM.

We therefore shifted our focus to analyzing the motifs at the amino acid level. Using WebLogos to visualize the relative frequencies of amino acids encoded in and around these nucleotide motifs, we identified several amino acids that appeared frequently in these plots. Specifically, leucine (L, 15.6%), phenylalanine (F, 11%), serine (S, 8%), and asparagine (N, 8%) were particularly prominent as amino acids that contained parts of the INM sequence (Figures 6A and 6B). This observation indicates that the INMs in HIV-1 *env* are biased toward encoding certain amino acids.

The prominence of L and S amino acids being encoded within the INMs raises intriguing questions about their potential role in viral evolution. Notably, certain L and S codons are known to occupy a “risky sequence space,” as they are only one mutation away from becoming stop codons (1-to-stop).^33^ Such mutations could lead to premature termination of protein translation, potentially disrupting protein (e.g., Env) function.^34^ Therefore, we investigated if T/F viruses have significant differences in their 1-to-stop codon usages compared to chronic viruses. If so, this could impose unique constraints on viral genome evolution, which might account for their reduced replicative fitness compared to the chronic viruses (Figure 5F). As shown, the number of “high-risk” leucine’s are significantly greater in T/F viruses as compared to chronic viruses (∗∗∗*p* ≤ 0.005) (Figure 6D). We also saw this with serine 1-to-stop codons (Figure 6E).

Given the significant differences in 1-to-stop codon usage between T/F acute and chronic viruses, we further analyzed whether codon usage biases were present for L and S codons in T/F viruses. Indeed, as shown in Figure 6C, we observed a bias in leucine codon usage. Specifically, T/F viruses exhibited elevated frequencies of TTA and CTC codons, while showing significant reductions in CTA and CTT codons. This interesting observation suggests how the “risky sequence space” occupied by L and S codons in T/F viruses may impose unique constraints on viral evolution. These findings also highlight the potential utility of genome screening for identifying T/F variants and also for identifying codon-level vulnerabilities, such as high-risk leucine and serine codons, that could influence viral fitness and evolution.

**Figure 7:**
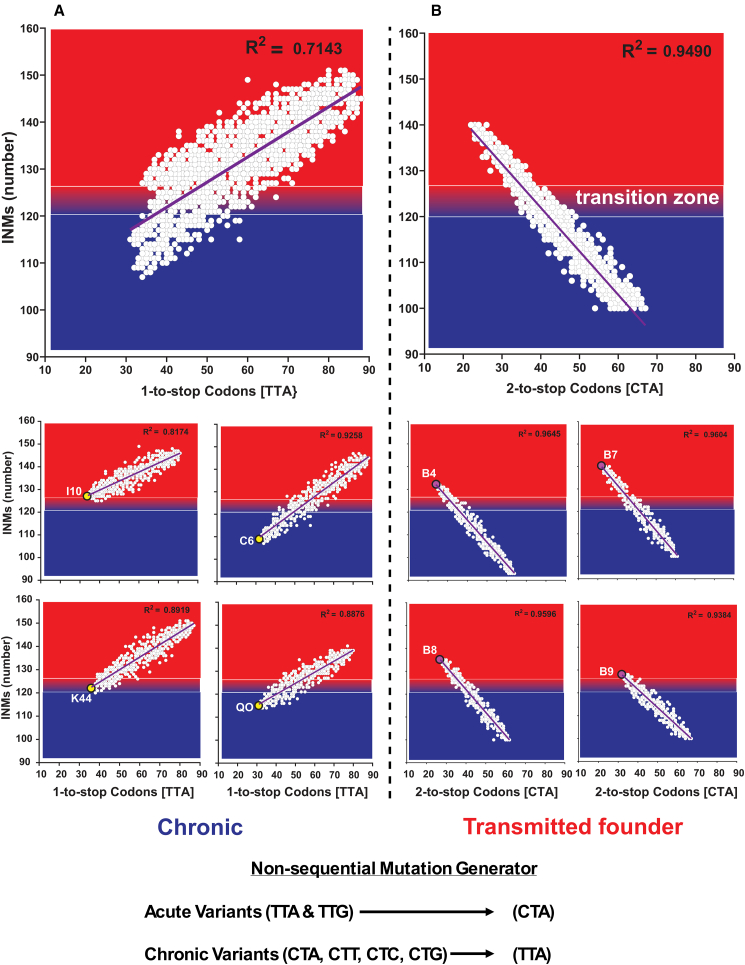
There is a reciprocal relationship that governs the presence of 1-to-stop codons and INMs in HIV In a systematic *in silico* analysis T/F (B4, B7, B9, B8) and chronic (I10, K44, Q0, C6) sequences were non-sequentially mutated to generate a set of variants each with a change in the underlying leucine codon frequency without modifying the total amount of leucine’s. (A) To model the evolution of T/F viruses toward chronic viral populations, high risk “1-to-stop” leucine codons (TTA, TTG) were mutated into “2-to-stop” codons (CTA) and the resulting number of INMs was quantified for each resulting variant. (B) In chronic viruses “2-to-stop” and “no-stop” (CTA, CTT, CTC, CTG) leucine codons were converted into “1-to-stop” (TTA) codons followed by quantification of INMs for each variant. The phenotype-mutation script was independently run 10 times with all results plotted relative to the original. Correlation between the increasing number of mutant leucine codons (TTA or CTA) and the correspondent change in INMs for each virus are presented.

Finally, building on the observation that INMs were enriched at leucine codons (Figure 6A), we investigated whether there is a sequence-level relationship between “high-risk” 1-to-stop leucine codons and INMs. To test this, we performed a systematic *in silico* analysis in which all leucine codons within the *env* sequences of eight HIV-1 viruses - T/F B4, B7, B8, B9, and chronic C6, I10, K44, Q0—were randomly mutated to alternative synonymous codons, preserving the amino acid sequence while varying nucleotide composition. By selectively introducing 1-to-stop leucine codons (TTA), we observed a positive correlation between the number of 1-to-stop codons and INM counts in chronic viruses (Figure 7A), suggesting these features are linked. To test the converse, we enriched T/Fs with “low-risk” 2-to-stop leucine codons at the expense of 1-to-stop codons. This exchange reduced INM abundance and shifted the T/F phenotype toward one more characteristic of chronic viruses (Figure 7B). Together, these findings indicate that the inflammatory potential encoded by INMs is specifically associated with 1-to-stop leucine codons, providing a plausible explanation for their enrichment in T/F viruses and underscoring their potential role in shaping early immune sensing of HIV-1.

## Discussion

Most of the work done on understanding HIV-1 transmission has focused on the phenotypic differences of the Env glycoprotein. These differences include changes in the glycosylation pattern, changes in the length of variable loops, increased Env density on the surface of the virions, and changes in co-receptor usage as well as entry efficiency (CCR5 to CXCR4).^2^^,^^9^^,^^10^^,^^35^^,^^36^ Yet it is still unclear what properties distinguish a true T/F virus from the rest of the quasi-species. It seems that beyond the use of CCR5,^2^^,^^9^ there is no single major phenotypic signature that is consistent for T/F. Rather, an array of potential phenotypic variations collectively, with undefined associations, is thought to enhance virus transmission, but even this can differ based on HIV-1 subtype responsible for infection. Our results describe an intriguing role for HIV-1 RNA, whereby the immunostimulatory potential of the viral RNA may enhance the sexual transmission of virus but may have a reduced role in establishment of chronic infection. These observations are consistent with antagonistic pleiotropy, in which context-dependent selection shapes distinct transmission and replication advantages.

The biological events during the earliest stages of HIV infection are only beginning to be understood, in part because virus sampling is limited to sources such as blood and semen from male donors, and the female genital tract and blood following penile-vaginal exposure. What is understood is that the viral inoculum into e.g., female genital tract, only establishes a few infectious foci in the submucosa layers within the first 3–4 days, as shown in non-human primate (NHP) studies.^37^ With regards to this work, at what time point viral RNA influences the transmission process can only be speculated upon; however, we believe it to be during early stages of genomic replication, when viral RNA and its transcripts are abundant and can be detected by host cell PRRs. It is known that migratory APC populations, such as DCs, play a significant role in capturing and transporting virus from mucosal tissues to local draining lymph nodes, and in endocytosing/phagocytosing virus and lysed cellular contents. Such APC populations express a range of viral RNA-sensing PRRs. Activation of TLR7/8 in these cells can trigger the release of cytokines such as IL-6, IL-1β, and IL-12, promoting Th1 and Th17 responses in mucosal tissues. Subsequent HIV-1 infection and spread through tissue-resident or tissue-homing T cells may then facilitate viral migration from mucosal sites to establish systemic infection.^38^^,^^39^^,^^40^ Accordingly, T/F viruses with elevated numbers of INMs may more effectively activate PRRs in mucosal DCs, triggering their maturation and migration from tissues, and thereby potentially enhance early viral dissemination into the systemic compartment. This could explain why we readily detected T/F acute viruses associated with migratory cells in our mucosal explant studies, and to a lesser extent chronic virus. It is plausible that selection occurs locally within small foci of infection, where only a limited number of cells, including CD4^+^ T cells and DCs, are initially involved. High-INM viruses may trigger sufficient local PRR activation to recruit additional target cells and amplify infection, whereas low-INM variants may fail to generate enough inflammation and undergo stochastic extinction. Under this model, early innate activation and viral amplification would be spatially restricted, allowing variant-specific effects to manifest before systemic spread occurs. In the future, it would be informative to see how viral RNAs containing different numbers of INMs modulates DC activation and migration.

The work presented here sheds important light upon within-host and between-host evolutionary dynamics. For instance, here we show that in addition to T/F/acute viruses having significantly increased transmission fitness relative to chronic-stage viruses, they exhibit significantly reduced replication fitness. This would suggest that over time, T/F viruses are replaced as the dominant variant by a quasi-species of rapidly diversifying viruses more adept to replicating in the new host. This is supported by our analysis of next-generation sequencing transcripts in mucosal explant assays, where we show chronic viruses replicated much better than T/F viruses. It was also shown by comparing T/F/acute and chronic virus replication in T cell lines and PBMC. Interestingly, it should be noted that viruses such as influenza, picornavirus, and HIV-1 have all previously been shown to exhibit reduced replication capacity when immunostimulatory sequence numbers are increased in their genomes.^41^^,^^42^^,^^43^^,^^44^^,^^45^ Therefore, given our data, it should be expected that T/F viruses with elevated numbers of immunostimulatory motifs display reduced replication fitness. This also implicates the ability of T/F viruses to exit genital tissues associated with migratory cells as being a key driver in the transmission fitness process, giving these variants the competitive advantage needed to establish a systemic infection. These observations align with models of HIV-1 evolution in which T/F viruses are optimized for transmission rather than maximal replication within the host. Rapid reversion of immune escape mutations to consensus sequences (“adapt and revert”) illustrates how viruses selected for transmission often carry a fitness cost in the new host.^46^^,^^47^^,^^48^ In parallel, the “archive and retrieve” model proposes that transmitted viruses resemble ancestral variants that were archived earlier in infection and subsequently emerge for transmission.^49^^,^^50^^,^^51^ Such variants may retain genomic features that favor transmission over within-host adaptation. While aspects of the work presented here are consistent with both models, the observed association between elevated INM content and transmission fitness is in line with predictions of the “archive and retrieve” model.

Given T/F viruses have increased INMs, it is plausible that T/F HIV-1 emerge out of the inoculating HIV-1 quasi-species based on their ability to induce immune activation within the recipient’s mucosa. This immune activation may be facilitated by a TLR7/8 response following endocytosis/phagocytosis of HIV by resident myeloid cells (macrophages, DCs, and Langerhan cells) in these tissues. In contrast, entry into T cells through plasma membrane/viral membrane fusion may induce more of a RIGI/cGAS response for intracellular innate immunity. Interestingly, a study on low dose influenza viral infection in mice, demonstrated that viral sensing through TLR7 or RIGI induces proinflammatory programs that promoted viral replication, potentially via a mechanism of immune target cell recruitment.^52^ The findings of Pang et al., 2013,^52^ have clear comparisons with the findings of this study.

This work also supports studies showing that the mucosal bottleneck selects for HIV-1 variants with increased resistance to antiviral IFN responses.^5^ Certainly, it makes sense that variants containing elevated numbers of INMs (i.e., viruses with elevated transmission fitness) would trigger innate immune responses more efficiently. Therefore, to counter the innate antiviral interferon response, only viruses with enhanced resistance would be selected for, i.e., T/F viruses. In the future, whether the antiviral IFN response triggered by T/F viruses adversely affects the viruses replicating in mucosal tissues will need to be investigated.

A particularly striking observation was an apparent compartmentalization of virus in semen compared to blood of HIV-1-infected individuals. Often, HIV-1 sequence diversity, and our understanding of viral biology is derived by studying viral reservoirs from blood. However, the data presented here suggests using viruses from blood could skew our understanding of T/F viruses, especially if blood-derived viruses are used in transmission studies. While the genotypic make-up of the virus in semen resembles that found in the blood, the numbers of INMs significantly differ. Testicular tissue is often referred to as an immunologically privileged site despite having leukocyte populations present.^53^ Whether there is indeed reduced immune pressure on the virus to evolve in this tissue will need to be investigated further, but it appears that virus in semen is a distinct reservoir and, based on our findings, probably more fit for transmission than that found in matched blood samples. A prior study evaluating HIV-1 diversity and genetic compartmentalization in blood and testes of antiretroviral therapy (ART)-suppressed individuals suggested that compartmentalization is present but attributed to clonal expansion of HIV-1-infected cells in the testes rather than separate levels of immune escape.^53^ Future studies should explore how HIV-1 virions in semen with high numbers of INMs compared to virions in blood with low numbers of INMs in their ability to infect and replicate in Th17 cells.

In summary, we have found an intriguing feature that is associated with and appears to successfully distinguish T/F viruses from the rest of the viral swarm. In so doing, we have also revealed important insights into the biology of HIV-1 transmission and provided supporting evidence for several previously published reports. We believe this work will help advance the understanding of HIV-1 transmission, and the information generated could be used to help rationally select and design the next generation of vaccine candidates, particularly those directed against T/F viruses.

### Limitations of the study

While the work presented here is intriguing, it is important to acknowledge certain caveats and limitations. Firstly, while this largely correlative analysis has only been applied to HIV-1, it will be important to see whether these findings are also observed in related retroviruses such as HIV-2 and simian immunodeficiency virus (SIV). Moreover, the impact of INMs numbers on viral transmission and disease progression has yet to be fully evaluated in a whole organism. For this, the SIV model, especially considering the differences in infection severity between old-world and new-world non-human primates, could provide valuable insights into HIV-1 transmission biology. Another limitation is the lack of access to published sequence data from transmission pairs that include blood samples from both the donor and recipient, along with matched genital secretion samples. Such datasets would enable the analysis of the full genetic bottleneck rather than relying on piecing together data from different cohorts, subtypes of infection, or varying lengths of genomic sequences. Additionally, whether the findings on INMs and transmission fitness translate into *in vivo* scenarios needs further exploration.

## Resource availability

### Lead contact

Requests for further information and resources should be directed to and will be fulfilled by the lead contact, Jamie F.S. Mann (jamie.mann@bristol.ac.uk).

### Materials availability

This study did not generate new unique reagents.

### Data and code availability

• This paper analyzes existing, publicly available data, accessible at:
  ○ Los Alamos National Laboratory (LANL) HIV Sequence Database: accession numbers: subtype A, GenBank: DQ676872, AB253421, AB253429, AF288623, GU201516; subtype B, GenBank: K03455, AY423387, AY173951, AY331295; subtype C, GenBank: U52953, U46016, AF067155, AY772699; subtype D, GenBank: K03454, AY371157, AY253311, U88824.
  ○ DOI: https://doi.org/10.1073/pnas.162014411; accession numbers: GenBank: KY111920–KY112584, KY364886.
  ○ DOI: https://doi.org/10.1371/journal.ppat.1006754; accession numbers GenBank: MF028814-F028921, MF028979-MF029088.
  ○ DOI: https://doi.org/10.1371/journal.ppat.1001053; accession numbers: GenBank: HM638460-HM639260.
• All original code has been deposited at GitHub and is publicly available at https://doi.org/10.5281/zenodo.20032602 as of the date of publication.
• Any additional information required to reanalyze the data reported in this paper will be made available from the lead contact upon request.

## Acknowledgments

This work was funded by awards from 10.13039/501100000024CIHR (PJT 149075) and MRC research grants (MR/Z504300/1) to J.F.S.M. and the NIH (AI49170) to E.J.A. We would like to thank the tissue donors from Charing Cross Hospital in London, United Kingdom.

## Author contributions

J.F.S.M. and K.K. designed the project; I.D., J.F.S.M., C.N.W., and M.A. wrote the python analysis scripts; R.P. and I.D. downloaded and performed the analysis of the various datasets; K.K. performed the tissue explant competition assay; R.P. and C.N.W. performed the T7 *in vitro* transcription and R.P. performed the THP-1 stimulation experiments. All listed authors contributed to writing, reviewing and editing the manuscript.

## Declaration of interests

The authors declare no competing interests.

## STAR★Methods

### Key resources table

**Table:** 

REAGENT or RESOURCE	SOURCE	IDENTIFIER
**Biological samples**		

Human penile tissue explants	This paper	Penile tissue obtained from people undergoing gender reassignment surgery

**Deposited data**		

HIV-1 group M reference genome sequences	Los Alamos National Laboratory HIV Database	Subtype A: DQ676872, AB253421, AB253429, AF288623, GU201516
HIV-1 group M reference genome sequences	Los Alamos National Laboratory HIV Database	Subtype B: K03455 (HXB2), AY423387, AY173951, AY331295
HIV-1 group M reference genome sequences	Los Alamos National Laboratory HIV Database	Subtype C: U52953, U46016, AF067155, AY772699
HIV-1 group M reference genome sequences	Los Alamos National Laboratory HIV Database	Subtype D: K03454, AY371157, AY253311, U88824
Panel of Full-Length T/F HIV-1 Infectious Molecular Clones	NIH AIDS Reagent Program	ARP-11919
Non-reference subtype B env sequences	Los Alamos National Laboratory HIV Database	*n* = 7,705
Subtype B env sequences	Center for HIV-1/AIDS Vaccine Immunology (CHAVI)	Study-derived sequence set

**Experimental models: Cell lines**		

HEK293T cells	ATCC — CRL-3216	Human embryonic kidney cell line
U87.CD4.CCR5 cells	BEI Resources/NIH HIV Reagent Program — ARP-4035.	HIV-1 target cell line
PM-1 CD4 T cells	BEI Resources/NIH HIV Reagent Program — ARP-3038.	Human CD4 T cell line
THP-1 Dual reporter cells	InvivoGen - thpd-nfis RRID:CVCL_X599	THP-1 Dual

**Oligonucleotides**		

ENVB primer	This paper	5′-AGAAAGAGCAGAAGACAG TGGCAATGA-3′
ED14 primer	This paper	5′-TCTTGCCTGGAGCTGCTT GATGCCCCAGAC-3′
E80 primer	This paper	5′-CCAATTCCCATACATTATTGTG-3′
E110 primer	This paper	5′-CTGTTAAATGGCAGTCTAGCAGAA-3′
E125 primer	This paper	5′-CAATTTCTGGGTCCCCTCCTGAGG-3′
T7_E80 primer	This paper	5′-TAATACGACTCACTATAGCCAATTC CCATACATTATTGTG-3′

**Recombinant DNA**		

pREC_nfl_NL4-3_Δenv/URA3	Dudley et al.^54^/previously described yeast-based recombination system	HIV-1 env recombination backbone
pCMV_cplt	Dudley et al.^54^/previously described yeast-based recombination system	Rescue plasmid

**Bacterial and virus strains**		

Acute env chimeric HIV-1 viruses	King et al.^21^	B1, B2, B3, B4, B7, B8, B9, B14, B17, B19, B20
Chronic env chimeric HIV-1 viruses	King et al.^21^	I10, K44, Q0

**Chemicals, peptides, and recombinant proteins**		

RPMI 1640 medium	Wisent	Cat.350-000-CL
Triton X-100	Millipore Sigma	Cat.T9284-100ML
Proteinase K	Thermo Fisher	Cat.4333793
PureLink™ *Pro* 96 Genomic DNA Purification Kit	Invitrogen / Thermo Fisher	Cat.K182104A
dNTP mix 10 mM	Thermo Fisher	Cat.FERR0192
Platinum™ *Taq* DNA Polymerase, High Fidelity	Thermo Fisher	Cat.11304029
Phusion™ High-Fidelity DNA Polymerase	Fisher Scientific	Cat. F530L
Phusion buffer	Fisher Scientific	Cat. F530L
MEGAscript™ T7 Transcription Kit	Thermo Fisher	Cat.AM1334
Dioleoyl-3-trimethylammonium propane (DOTAP)	Millipore Sigma	Cat.11202375001
Agencourt AMPure XP beads	Beckman Coulter	Cat.NC9959336
Quant-iT PicoGreen dsDNA assay	Thermo Fisher	Cat.P7589

**Software and algorithms**		

ClustalW	BioEdit	–
BioEdit	BioEdit	–
PyCharm Community Edition	JetBrains	Community Edition
GraphPad Prism	GraphPad Software	Version 10 for motif analyses; Prism also used for statistics
SeekDeep	Reference 5 / custom pipeline	Barcode sorting and within-bin sequence alignment
Graphreader	graphreader.com	Web-based figure digitization tool
Original python script	GitHub	https://doi.org/10.5281/zenodo.20032602

**Other**		

Roche 454 GS Junior sequencer	Roche	GS Junior (discontinued)
Cytation 5 plate reader	Agilent BioTek	Cytation 5

### Experimental model and study participant details

#### Human tissue samples

Human penile tissue was obtained from people undergoing gender reassignment surgery. Tissue collection was performed with written informed consent according to local research committee guidelines. All tissues were collected under protocols approved by the National Research Ethics Committee in accordance with the Human Tissue Act 2004.

#### Cell lines

HEK293T cells were used for production of Env chimeric HIV-1 virus stocks. U87.CD4.CCR5 cells were used for virus propagation. PM-1 CD4 T cells were used for co-culture following tissue explant infection. THP-1 Dual reporter cells were used for *env* RNA stimulation assays. Cell line identifiers and sources are provided in the key resources table.

### Method details

#### DNA sequences

HIV-1 sequences used in this study were obtained from previously published cohort studies.^5^^,^^22^^,^^23^^,^^30^ Whole-genome sequences for representative HIV-1 group M reference strains from subtypes A, B, C, and D were downloaded from the Los Alamos National Laboratory (LANL) HIV Sequence Database. Reference accessions were as follows: subtype A, DQ676872, AB253421, AB253429, AF288623, and GU201516; subtype B, K03455, AY423387, AY173951, and AY331295; subtype C, U52953, U46016, AF067155, and AY772699; and subtype D, K03454, AY371157, AY253311, and U88824. In addition, 8,040 non-reference subtype B *env* sequences were downloaded from LANL. *env* sequences from the Panel of Full-Length Transmitted/Founder HIV-1 Infectious Molecular Clones were obtained through the NIH AIDS Reagent Program (ARP-11919), and additional subtype B *env* sequences were obtained from the Center for HIV/AIDS Vaccine Immunology (CHAVI).

#### Sequence alignment and preprocessing

For analyses involving full-length HIV-1 genomes, reference sequences were aligned to HXB2 using the ClustalW algorithm. Genomic regions corresponding to *gag* (HXB2 positions 790–2292), *pol* (2085–5096), *env* (6225–8795), *vif-vpr-5′tat-vpu* (5041–6310), and *nef* (8797–9417) were then trimmed and de-gapped in BioEdit. For analyses restricted to *env*, sequences were aligned to HXB2 *env*, trimmed to the region of interest, and de-gapped prior to downstream analysis.

Before trimming, aligned *env* sequences were manually reviewed to confirm complete coverage across the region being analyzed. Sequences lacking full *env* coverage were excluded. For comparisons involving cervical genital secretion- and blood-derived recipient sequences, only partial *env* regions were available from the original studies. These sequences were therefore aligned to HXB2 and trimmed to the shared C2–V5 or C2–V3 interval, respectively, such that all sequences within a given analysis were of equal length.

#### *In silico* synonymous leucine codon mutagenesis

To examine the relationship between synonymous leucine codon usage and immunostimulatory nucleotide motif abundance, an in-house Python script was used to systematically replace leucine codons within HIV-1 *env* with synonymous codons classified as either high-risk or low-risk. High-risk leucine codons were defined as TTA and TTG, whereas low-risk leucine codons were defined as CTG, CTC, CTA, and CTT. All high-risk and low-risk codon classes were first enumerated within each parental *env* sequence.

Starting from the original *env* sequence for each new variant, the script randomly and non-sequentially replaced full leucine codons (3 nt) to progressively shift the sequence toward either a high-risk or low-risk leucine codon composition. For high-risk conversion, low-risk codons were replaced with TTA or TTG. For low-risk conversion, high-risk codons were replaced with low-risk synonymous codons which were: CTG, CTA, CTT or CTC. At each iteration, the number of mutated leucine codons increased relative to the parental sequence, and each intermediate sequence was logged and aligned back to the original sequence. The final sequence variant in each run contained only high-risk or only low-risk leucine codons at leucine positions within *env*.

This mutational progression was performed independently 10 times for each parental *env* sequence (B4, B7, B8, B9, K44, Q0, C6, and I10). The resulting aligned sequence sets were exported in FASTA format and used for enumeration of immunostimulatory motifs. The relationship between the number of high-risk or low-risk leucine codons and the total number of immunostimulatory nucleotide motifs per variant was then analyzed by simple linear regression in GraphPad Prism.

#### Identification of immunostimulatory nucleotide motifs

To quantify sequence motifs associated with innate immune stimulation, an in-house Python script was developed and run in PyCharm Community Edition. The script scanned FASTA- and GENBANK-formatted sequences for the presence of 12 previously defined immunostimulatory motifs: TTGT, TTTC, TGTT, CTGT, TATT, TTTA, TTGC, GTTT, ATTT, ATGT, CTTT, and TCTT. CpG dinucleotides were also enumerated. Motif counts generated by the script were compiled for downstream analysis and visualization in GraphPad Prism 10.

#### Generation of chimeric viruses

Eleven early/acute subtype B envelopes were obtained from the Center of HIV-1/AIDS Vaccine Immunology (CHAVI).^2^ Three chronic subtype B envelopes were obtained from a Belgian cohort.^21^^,^^55^ Chimeric viruses were created using a previously established yeast-based recombination system.^56^ Briefly, patient-derived *env* sequences were RT-PCR amplified and co-transformed into yeast together with linearized *pREC_nfl_NL4-3_Δenv/URA3*, allowing homologous recombination-mediated replacement of the *URA3* cassette with the corresponding *env* insert. Recombinant yeast colonies were selected on C-LEU/5-FOA plates and screened by PCR for the presence of full-length *env* inserts. Plasmids recovered from positive clones were purified and co-transfected into HEK293T cells with the complementing rescue plasmid *pCMV_cplt* to generate infectious virus, which was subsequently amplified in U87.CD4.CCR5 cells. As a result, the pREC constructs differed solely on the inserted *env* sequence. The acute viruses are referred to as: B1, B2, B3, B4, B7. B8, B9. B14. B17, B19 and B20. The chronic viruses are referred to as: I10, K44 and Q0. RT activity of each virus was used to quantify infectivity for future tissue infections.

#### Tissue explant model studies

Penile tissue was obtained from men undergoing gender reassignment surgery and sectioned into approximately 3 mm × 3 mm × 3 mm explants. For each condition, 3-5 tissue pieces were placed into individual wells of a 48-well plate. Pools containing four to five acute or three chronic Env chimeric viruses (150 normalized virus units per virus, based on RT activity) were added to culture medium containing 3-5 explants and incubated for 3 h at 37°C. Following incubation, explants were washed three times with complete RPMI 1640 medium (Sigma). Migratory cells (MCs) released from the tissue were harvested at 24 h post-infection and co-cultured with 1 × 10ˆ5 PM-1 CD4 T cells. On day 10, tissue explants were lysed in 300 μL 1% Triton X-100 supplemented with proteinase K and incubated overnight at 56°C, while cells from the MC-PM-1 co-cultures were lysed in 300 μL 1% Triton X-100. Genomic DNA was extracted using the PureLink Pro 96 Genomic DNA kit (Invitrogen) according to the manufacturer’s instructions. Each multi-virus competition assay was performed using tissue from two donors, with each condition tested in triplicate.

The C2-V3 region of HIV-1 *env* was amplified using a two-step external nested PCR approach. External PCR amplification was performed using primers ENVB (5′AGAAAGAGCAGAAGACAGTGGCAATGA-3′) and ED14 (5′-TCTTGCCTGGAGCTGCTTGATGCCCCAGAC-3′). Nested PCR amplification was performed using primers E80 (5′-CCAATTCCCATACATTATTGTG-3′) and E125 (5′-CAATTTCTGGGTCCCCTCCTGAGG-3′). Each PCR reaction contained 0.2 μM of each primer, 1.5 mM MgCl_2_, 1× Platinum Taq buffer, 0.2 mM dNTPs, and 2 U Platinum Taq DNA polymerase. Cycling conditions consisted of an initial denaturation at 95°C for 2 min, followed by 35 cycles of 95°C for 30 s, 55°C for 30 s, and 72°C for 2 min for the external PCR or 45 s for the nested PCR, with a final extension at 72°C for 10 min.

Amplicon libraries were generated using fusion primers containing Roche 454 Titanium key sequences and multiplex identifier (MID) barcodes, followed by template-specific sequences corresponding to forward primer E110 (5′-CTGTTAAATGGCAGTCTAGCAGAA-3′) and reverse primer E125 (5′-CAATTTCTGGGTCCCCTCCTGAGG-3′). Amplification was performed using the same reaction conditions described above, with an extension time of 45 s. PCR products were resolved on a 1% agarose gel to confirm the expected amplicon size (406 bp) and purified using Agencourt AMPure XP beads at a 0.7:1 bead-to-DNA ratio. Libraries were quantified using the Quant-iT PicoGreen dsDNA assay, normalized, and pooled at 10ˆ6 molecules/μL. Emulsion PCR was then performed at a ratio of 0.5 library molecules per bead. Enriched beads (5 × 10ˆ5) were loaded onto Titanium picotiter plates and sequenced on a Roche 454 GS Junior instrument according to the manufacturer’s instructions. Sequence reads were processed using SeekDeep, a custom pipeline^6^ that sorts sequences based on barcode identity and aligns sequences within barcode bins, and viruses within each competition were quantified from these outputs.

#### Env Antigenicity

The *env* gene from B4, B7 and B9 (acute/early) and Q0, K44, I10 (chronic) viruses were amplified using primers T7_E80 (TAATACGACTCACTATAGCCAATTCCCATACATTATTGTG) and E125 (CAATTTCTGGGTCCCCTCCTGAGG). The following PCR cycling conditions were used: cycle 1 consisted of a 98°C step for 2 minutes; cycle 2 consisted of 3 stages repeated 10 times and consisted of a 98°C step for 10 seconds, followed by a 55°C step for 30 seconds, and then 72°C for 8 minutes; cycle 3 consisted of 3 stages repeated 25 times at 98°C for 10 seconds, followed by 55°C for 30 seconds and finally 78°C for 8 minutes. Lastly, cycle 4 consisted of a 72°C step for 7 minutes before incubation at 4°C. The PCR amplification reaction consisted of: 1X Phusion buffer (Thermo Fisher), 10 μM of dNTP mixture (Thermo Fisher), 1 U of Phusion DNA polymerase (Thermo Fisher), 0.5 μM of forward and reverse primers.

Following gel extraction, the amplicons were used for downstream *in vitro* transcription using a T7 MEGAscript™ transcription kit (ThermoFisher) according to the manufacturer’s instructions. Briefly, 2 μL of each ribonucleotide, 2 μL of 10X reaction buffer, 0.15 μg of PCR product, 2 μL of T7 enzyme mix and DNA/RNA free water (Sigma) was added to yield a total reaction volume of 20 μL. The reaction was incubated at 37°C for 16 hours in a PCR Thermocycler to increase number of transcription initiation events.

To test the antigenicity of the various *env* RNA, THP-1 Dual™ reporter cells (Invivogen, USA) were seeded 1 × 10ˆ5 cells/well in a 96-well plate and stimulated with 2.5 μg of *env* RNA complexed with 5 μg of dioleoyl-3-trimethylammonium propane (DOTAP) (Roche). Prior to stimulation, the RNA: DOTAP complex was incubated in incomplete RPMI for 15 minutes at room temperature. Cells were cultured with the stimulant for 24 hours at 37°C and 5% CO_2_ before assessing the supernatant for IRF (via detection of secreted luciferase) and NF-kB (via detection of secreted embryonic alkaline phosphatase (SEAP) activation according to manufacturer’s instructions. A Cytation5 reader was used to measure absorbance readings at 630 nm. Four independent stimulations were performed for the measure of IRF and NF-kB activation.

#### Pseudovirus replication fitness analysis

Data from King et al.*,*^21^ examining the replication of eleven acute subtype B Env pseudoviruses (Envs from Center for HIV/AIDS Vaccine Immunology (CHAVI) acute infection studies^2^ and 14 chronic Envs (isolated from patients infected for more than 1 year (Case Western Reserve University^21^) in PM1 and PBMC were extracted using graphreader.com (http://www.graphreader.com/).

### Quantification and statistical analysis

Linear regression analyses were performed in GraphPad Prism as indicated in the corresponding figure legends. Graphs were generated in Prism from motif counts and codon-substitution outputs produced by the in-house Python pipeline. Where two groups are being assessed, statistical analysis of the data was carried out using a Mann-Whitney non-parametric *t* test (unpaired) GraphPad PRISM software.
