# Supplementary material for: The genomic sequence of packaged viral ribonucleic acid predicts mucosal HIV-1 transmission fitness
Source: iScience. 2026 Jul 13;29(8):116712. doi: 10.1016/j.isci.2026.116712 (PMC13382436; doi:10.1016/j.isci.2026.116712)
Supplement: Document S1. Figures S1–S4 [file mmc1.pdf]

iScience, Volume *29*

Supplemental information

The genomic sequence of packaged viral ribonucleic acid predicts mucosal HIV-1 transmission fitness

Rahul Pawa, Iulian Derecichei, Katja Klein, Muhammad Ali, Chanuka N. Wijewardhana, Richard Gibson, Ryan M. Troyer, Robin J. Shattock, Eric J. Arts, and Jamie F.S. Mann

Figure S1: ***Published clinical datasets representing a range along the genetic bottleneck.*** All the clinical datasets utilized in this study, superimposed over the idealized timeline of heterosexual HIV-1 infection is shown.

Figure S2: ***For clades A, B, C and D, env displays the highest prevalence of INMs.*** Whole genome sequences for the reference strains of each subtype/clade (A, B, C, D, E, F, G, H) were downloaded from the Los Alamos National Laboratory HIV database (https://www.hiv.lanl.gov). The genome was divided into 5 parts*: gag, pol, env, vif-vpr-5'tat-vpu,* and *nef* based on HXB2 alignment. Graphs display the numbers and percent of INMs and numbers and percent of CpG motifs for **(A)** all combined subtypes, **(B)** subtype A, **(C)** subtype B, **(D)** subtype C and **(E)** subtype D.

Figure S3: ***The recipients within the Swiss Cohort transmission pairs have viral genomic sequences with higher numbers of immunostimulatory RNA motifs compared to their respective donors.*** Donor and recipient HIV-1 sequences, attained from [S1] were aligned based on HXB2 and trimmed such that all *env* sequences were the same length. All donor and recipient sequences were analyzed for the (**A**) numbers and (**B**) percent of INMs and (**C**) numbers and (**D**) percent of CpG motifs. Statistical significance was assessed using a non-parametric Mann-Whitney test for comparisons of mean rank values among the different groups (**$p \leq 0.05$, ****$p \leq 0.0005$).

Figure S4: ***HIV C2-V3 region trimmed analysis of all published datasets.*** All the clinical datasets utilized in this study trimmed to C2-V3 to match the analysis in the Klein *et al.*, 2018 [S2] dataset. Sequences were analyzed for the numbers and percent of INMs and numbers and percent of CpG motifs. Statistical significance was assessed using a non-parametric Mann-Whitney test for comparisons of mean rank values among the different groups

(*p ≤ 0.05, ** *p* ≤ 0.005, *****p* ≤ 0.0005). Data for INMs and CpG motifs represented as both, number and percentage of genome.

Figure S1.

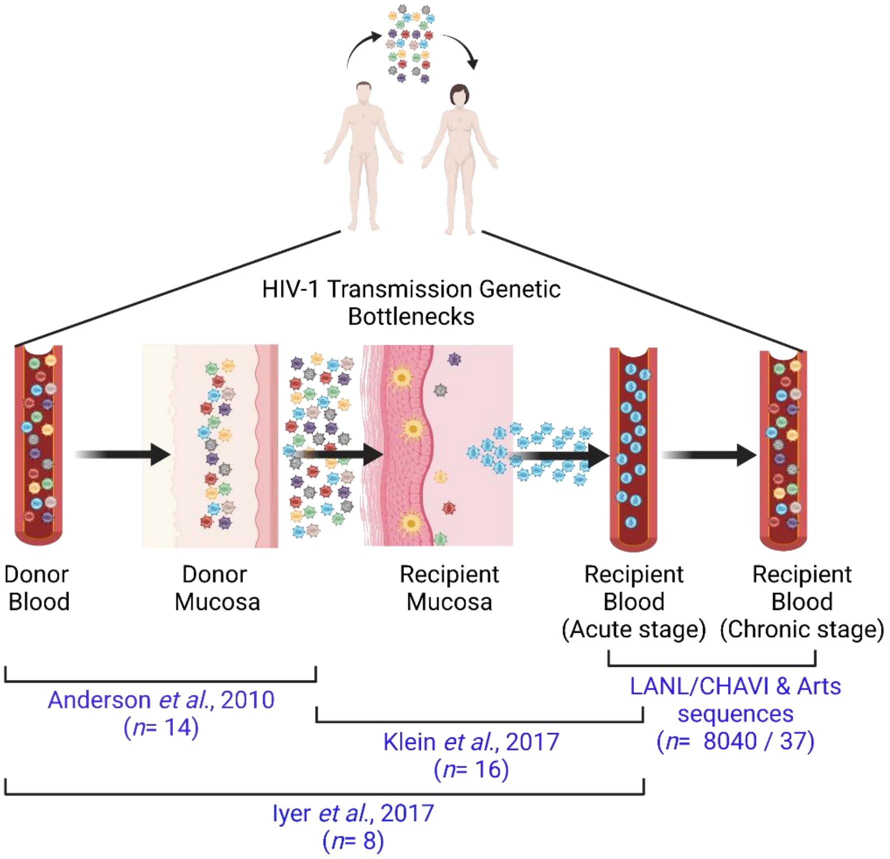
HIV-1 Transmission Genetic Bottlenecks
Donor Blood
Donor Mucosa
Recipient Mucosa
Recipient Blood (Acute stage)
Recipient Blood (Chronic stage)
Anderson et al., 2010 (n= 14)
Klein et al., 2017 (n= 16)
LANL/CHAVI & Arts sequences (n= 8040 / 37)
Iyer et al., 2017 (n= 8)

Figure S2.

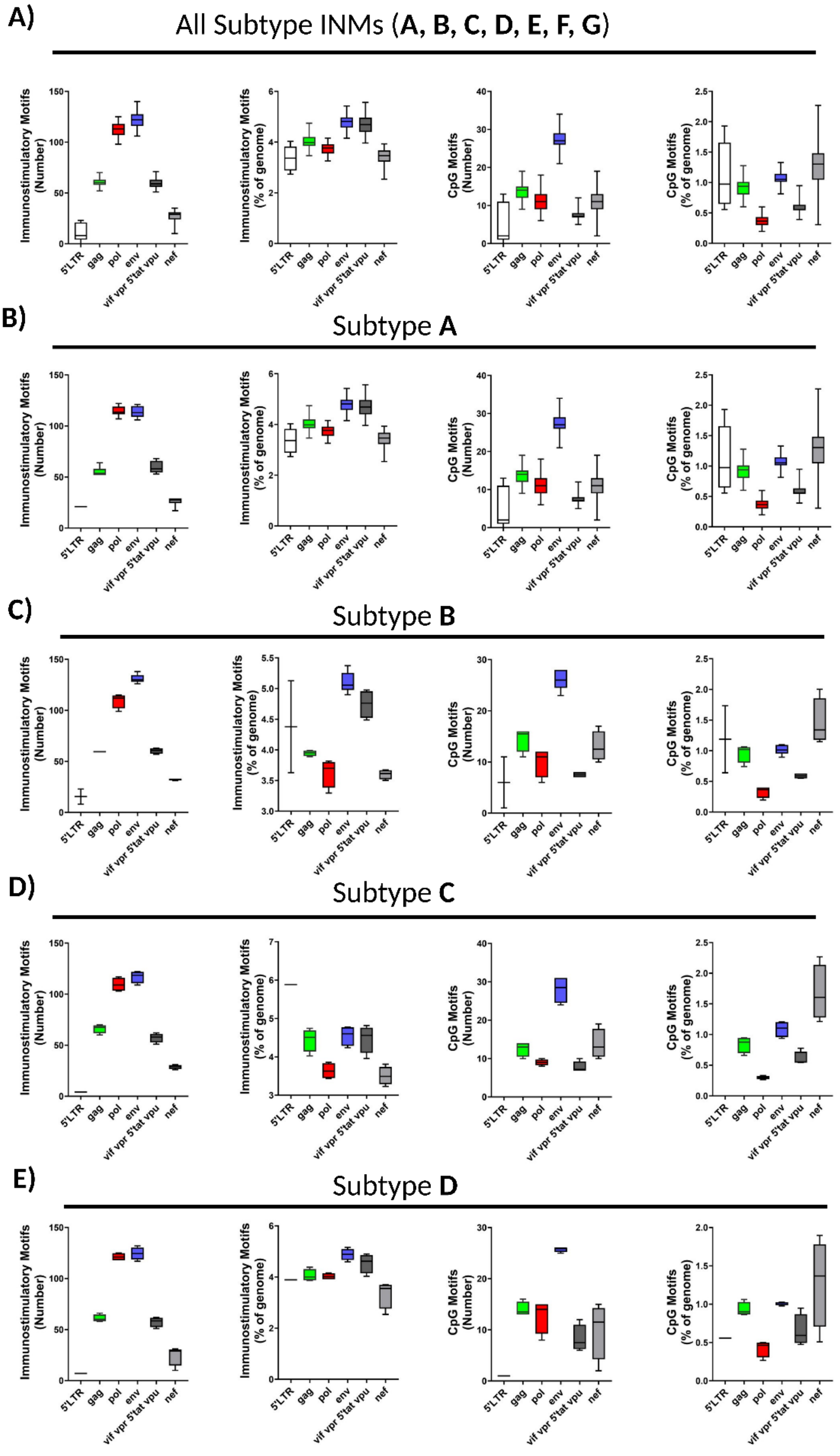

A)
All Subtype INMs (A, B, C, D, E, F, G)
B)
Subtype A
C)
Subtype B
D)
Subtype C
E)
Subtype D
Immunostimulatory Motifs (Number)
Immunostimulatory Motifs (% of genome)
CpG Motifs (Number)
CpG Motifs (% of genome)
5'LTR
gag
pol
env
vif vpr 5'tat vpu
nef

Figure S3.

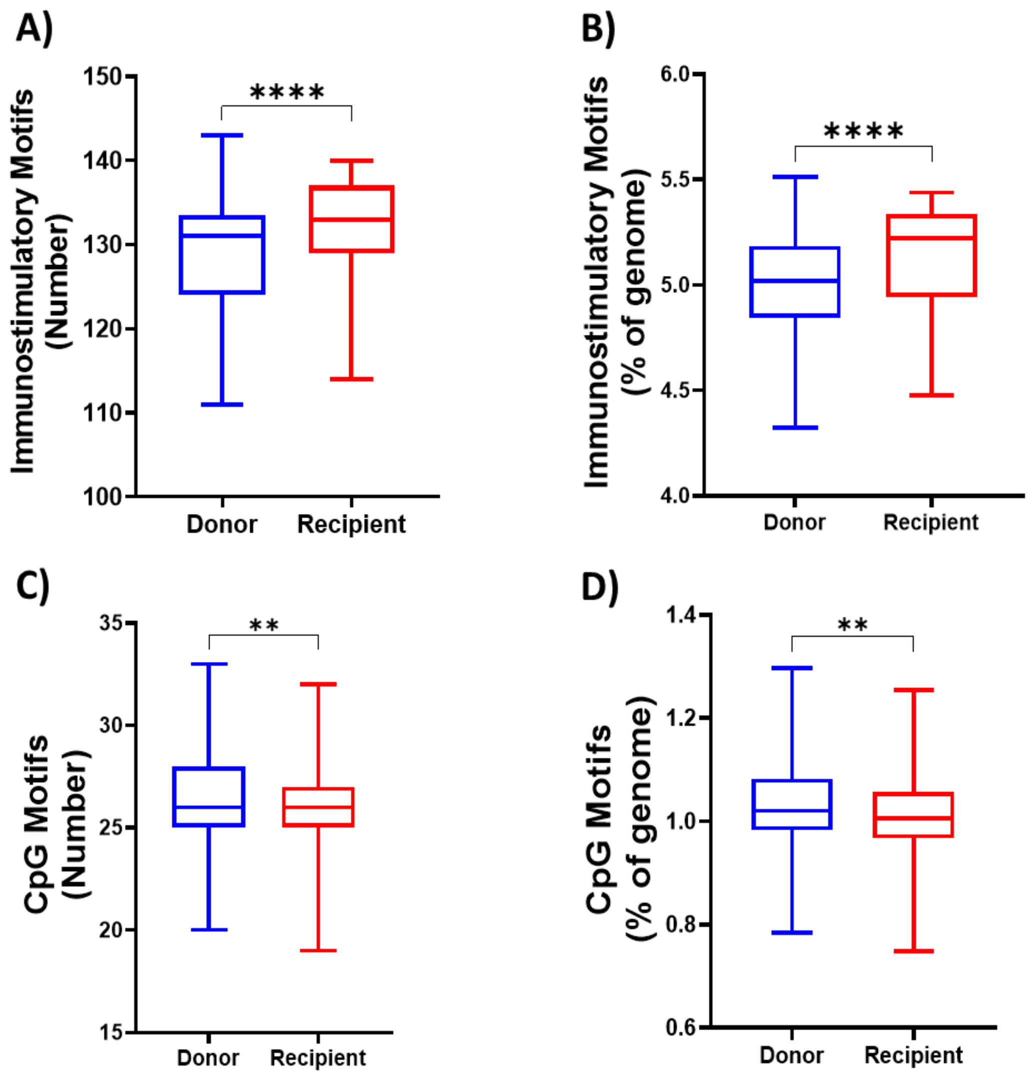

A)
Immunostimulatory Motifs (Number)
150
140
130
120
110
100
****
Donor
Recipient
B)
Immunostimulatory Motifs (% of genome)
6.0
5.5
5.0
4.5
4.0
****
Donor
Recipient
C)
CpG Motifs (Number)
35
30
25
20
15
**
Donor
Recipient
D)
CpG Motifs (% of genome)
1.4
1.2
1.0
0.8
0.6
**
Donor
Recipient

Figure S4.

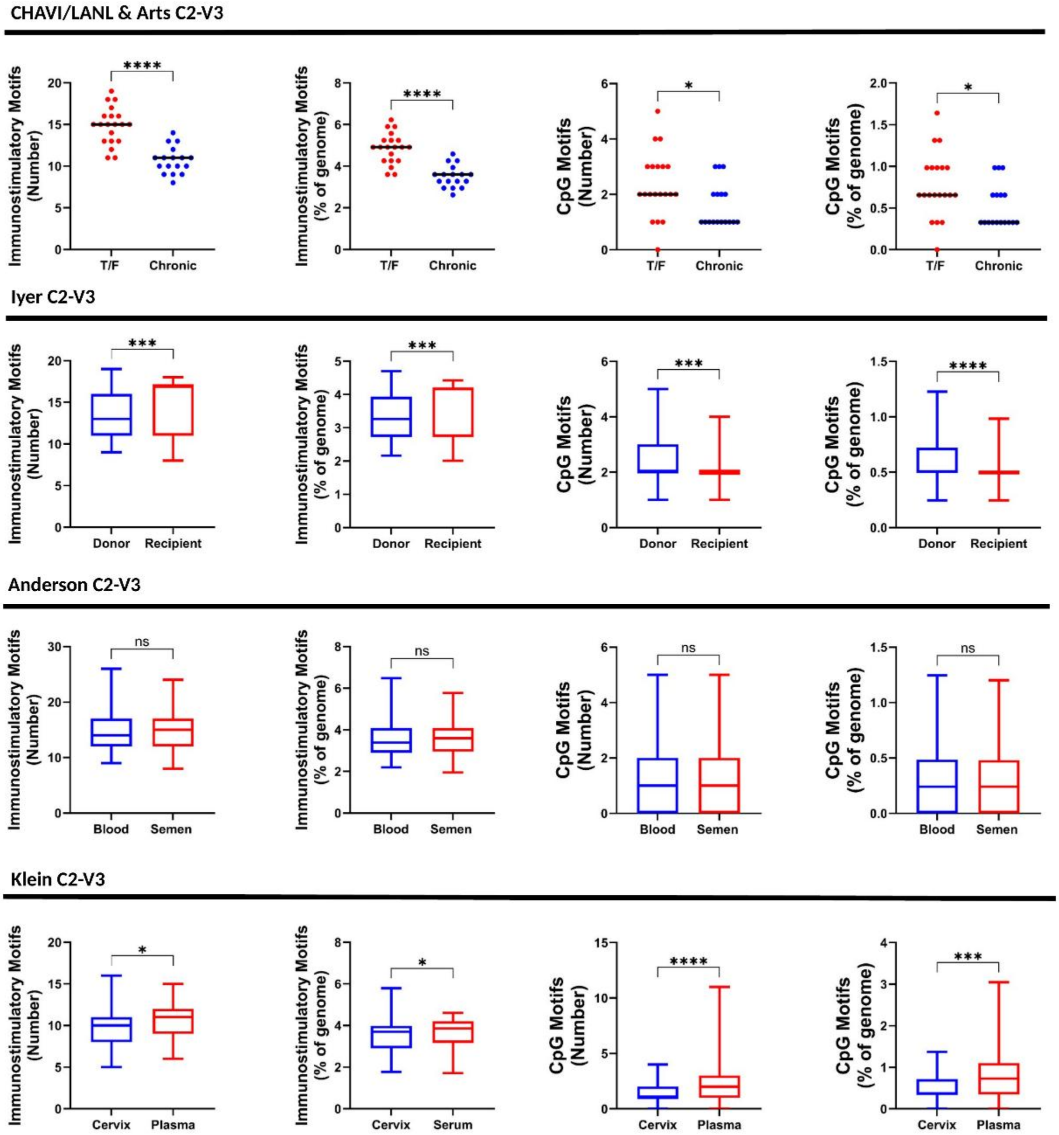

CHAVI/LANL & Arts C2-V3
Immunostimulatory Motifs (Number)
Immunostimulatory Motifs (% of genome)
CpG Motifs (Number)
CpG Motifs (% of genome)
T/F
Chronic
****
*
Iyer C2-V3
Donor
Recipient
***
****
Anderson C2-V3
Blood
Semen
ns
Klein C2-V3
Cervix
Plasma
Serum

References:

1. Oberle, C.S., Joos, B., Rusert, P., Campbell, N.K., Beauparlant, D., Kuster, H., Weber, J., Schenkel, C.D., Scherrer, A.U., Magnus, C., et al. (2016). Tracing HIV-1 transmission: envelope traits of HIV-1 transmitter and recipient pairs. Retrovirology *13*, 62. 10.1186/s12977-016-0299-0.
2. Klein, K., Nickel, G., Nankya, I., Kyeyune, F., Demers, K., Ndashimye, E., Kwok, C., Chen, P.L., Rwambuya, S., Poon, A., et al. (2018). Higher sequence diversity in the vaginal tract than in blood at early HIV-1 infection. PLoS Pathog *14*, e1006754. 10.1371/journal.ppat.1006754.
